# 25CN-NBOMe Metabolites in Rat Urine, Human Liver Microsomes and *C. elegans—*Structure Determination and Synthesis of the Most Abundant Metabolites

**DOI:** 10.3390/metabo11040212

**Published:** 2021-03-31

**Authors:** Anna Šuláková, Jitka Nykodemová, Petr Palivec, Radek Jurok, Silvie Rimpelová, Tereza Leonhardt, Klára Šíchová, Tomáš Páleníček, Martin Kuchař

**Affiliations:** 1 Department of Experimental Neurobiology, National Institute of Mental Health, Topolová 748, 250 67 Klecany, Czech Republic; anna.sulakova@nudz.cz (A.Š.); klara.sichova@nudz.cz (K.Š.); tomas.palenicek@nudz.cz (T.P.); 2 Forensic Laboratory of Biologically Active Substances, Department of Chemistry of Natural Compounds, University of Chemistry and Technology Prague, Technická 5, 16 628 Prague, Czech Republic; nykodemj@vscht.cz (J.N.); palivecp@vscht.cz (P.P.); 3 Department of Organic Chemistry, University of Chemistry and Technology Prague, Technická 5, 16 628 Prague, Czech Republic; jurokr@vscht.cz; 4 Department of Biochemistry and Microbiology, University of Chemistry and Technology Prague, Technická 5, 16 628 Prague, Czech Republic; rimpelos@vscht.cz (S.R.); pikalovt@vscht.cz (T.L.)

**Keywords:** 25CN-NBOMe, LC-MS, metabolite synthesis, metabolomics

## Abstract

*N*-Benzylphenethylamines are novel psychedelic substances increasingly used for research, diagnostic, or recreational purposes. To date, only a few metabolism studies have been conducted for *N*-2-methoxybenzylated compounds (NBOMes). Thus, the available 2,5-dimethoxy-4-(2-((2-methoxybenzyl)amino)ethyl)benzonitrile (25CN-NBOMe) metabolism data are limited. Herein, we investigated the metabolic profile of 25CN-NBOMe in vivo in rats and in vitro in *Cunninghamella elegans* (*C. elegans*) mycelium and human liver microsomes. Phase I and phase II metabolites were first detected in an untargeted screening, followed by liquid chromatography-tandem mass spectrometry (LC-MS/MS) identification of the most abundant metabolites by comparison with in-house synthesized reference materials. The major metabolic pathways described within this study (mono- and *bis-O-*demethylation, hydroxylation at different positions, and combinations thereof, followed by the glucuronidation, sulfation, and/or *N*-acetylation of primary metabolites) generally correspond to the results of previously reported metabolism of several other NBOMes. The cyano functional group was either hydrolyzed to the respective amide or carboxylic acid or remained untouched. Differences between species should be taken into account in studies of the metabolism of novel substances.

## 1. Introduction

2,5-Dimethoxy-4-(2-((2-methoxybenzyl)amino)ethyl)benzonitrile (25CN-NBOMe) is a novel psychoactive substance (NPS) belonging to the extensive *N*-2-methoxybenzyl (NBOMe) family, the structure of which emerges from the renowned 2,5-dimethoxyphenethylamines functionalized at the 4-position (2C-X).

The unprecedented and intriguing discovery that benzylation of the aminic nitrogen of 2C-X can lead to potently biologically active compounds [1] opened a broad scene for expansive, hitherto unexplored realms in the world of psychoactive drugs, and inevitably prompted comprehensive research endeavors investigating plethora analogs.

Efforts in the pursuit of novel diagnostic, therapeutic, and research tools resulted in several notable implementations of *N*-benzylphenethylamines (NBPEAs), such as utilization of 2-(4-bromo-2,5-dimethoxyphenyl)-*N*-(2-methoxybenzyl)ethan-1-amine (25B-NBOMe) as a positron emission tomography (PET) radiolabeled tracer known as [^11^C]Cimbi-36, which has been used for visualization and quantification of serotonin 2A (5-HT2A) receptor in the human brain [2,3], or the discovery of 4-(2-((2-hydroxybenzyl)amino)ethyl)-2,5-dimethoxybenzonitrile (25CN-NBOH) as one of the most selective agonist ligands for the 5-HT2A receptor known to date [4,5,6].

Explorational research has been thoroughly carried out also behind the doors of academic and clinical laboratories, spawning spates of novel analogs of the immense theoretical structural variety, often not yet recorded in literature. Some of them materialized, flooding the NPS market since 2010 [7,8], and NBPEAs are now well-established street drugs at many places around the world [9].

Due to their current potential uses in many domains of interest, pharmacological and toxicological characterizations of NBPEAs, as well as an understanding of their metabolism, are necessary.

In the past, a significant correlation between 5-HT2 receptor affinity and potency of several hallucinogenic substances in rats was observed [10,11]. Currently, agonism at the 5-HT2A receptor is a major factor believed to be important and implicated in mediating the subjective and mind-altering effects of a drug of the “classical psychedelic” classes [12,13,14,15]. Since *N*-benzylation of 2C-X compounds dramatically enhances their 5-HT2A receptor affinity and improves selectivity over other 5-HT receptor subtypes [1,16,17,18,19,20,21], NBPEAs represent an intriguing investigational aim. Compared to classical 2C-X compounds, NBOMes often exhibit subnanomolar affinities for the 5-HT2 receptors, along with increased binding affinity at serotonergic 5-HT2A, 5-HT2C, adrenergic alpha1, dopaminergic D1–3, and histaminergic H1 receptors, monoamine transporters, and reduced binding to 5-HT1A receptors and trace amine-associated receptor 1 (TAAR1) [21]. However, NBOMes are substantially weaker in functional assays at the 5-HT2A receptor than their 2C-X counterparts [20]. The binding profiles of NBPEAs predict strong hallucinogenic effects in humans similar to lysergic acid diethylamide (LSD), possibly with increased stimulant action due to alpha1 adrenergic interactions [21]. Head twitch response (HTR) assays in mice, currently widely used laboratory animal behavioral proxy for 5-HT2A receptor-mediated hallucinogenic effects in humans [22,23,24], have also shown high potency of NBPEAs [25,26]. The in vitro characteristics and animal behavioral assays correlate with their potency known from their recreational uses [9]. The narrow pharmacological-toxicological window makes NBPEAs potentially dangerous substances if mistaken for other relatively safe drugs such as LSD [27,28]. In overdose amounts, NBOMes have been anecdotally reported to occasion acute sympathomimetic and cardiovascular toxicity [9].

Findings from different metabolic studies of 25I-NBOMe [29,30,31], 25B-NBOMe, and 25C-NBOMe [32] thus far tentatively indicate similarities in the extensive metabolic pathways of NBOMe compounds. Primary metabolites are 5-*O*-desmethyl-25X-NBOMe, 2-*O*-desmethyl-25X-NBOMe, 25X-NBOH, and their glucuronic acid conjugates, along with a large array of minor metabolites, such as *N*-debenzylation (2C-X), or ring- or chain-hydroxylation [29,30,31,33,34,35,36,37,38,39,40,41]. The major cytochrome P450s responsible for biotransformation of these compounds have been identified as CYP3A4, CYP2C9, CYP2C19, CYP1A2, CYP2B6 [33,42].

The average intrinsic clearance is higher for NBOMes compared to 2C-X (6.0 [1.9–14.0] and 0.51 L/kg/h, respectively), predictive of high first-pass metabolism in the liver [36]. These findings correlate with their lack of peroral bioavailability—the most commonly employed routes of administration of powdered or liquid NBOMes being sublingual, buccal, or intranasal [43].

The synthesis of 25CN-NBOMe was first published in the literature in 2010 among a series of tenths of compounds with structural variations, determining their effects on receptor binding affinities and functional activities at 5-HT2A and 5-HT2C receptors [4,5].

Besides these preliminary in vitro characteristics, no data are available on the animal and human absorption, distribution, metabolism, and excretion (ADME) properties of 25CN-NBOMe, partly due to the ethical and legal concerns associated with their use. In the light of the increasing use of NBOMes in research and recreation, there is an urgent need for controlled experiments identifying the pharmacological properties, identifying metabolites, and establishing potential drug-drug interactions of this novel compound.

Here, we present a study establishing the in vitro and in vivo metabolism of 25CN-NBOMe, analyzing its phase I and phase II metabolites in rats, human liver microsomes (HLM), and *Cunninghamella elegans* (*C. elegans*) fungus mycelium with the confirmation of the most abundant metabolites by the synthesis of their reference standards.

## 2. Results and Discussion

### 2.1. Untargeted Screening and Detection Of Metabolites

First, an untargeted screening approach was applied to predict major metabolic pathways. Rat urine, human liver microsomes, and *C. elegans* culture medium samples were separated using reversed-phase high-performance liquid chromatography (RP HPLC), and both high-resolution mass spectrometry (HR-MS) only and high-resolution tandem mass spectrometry (HR-MS/MS) measurements were performed by a triple quadrupole time of flight instrument. 25CN-NBOMe phase I and II metabolites were identified based on the precursor ion exact masses (PM) and most abundant fragment ions (FI) in HR-MS/MS. All detected metabolites are summarized in Table 1.

Altogether twenty-seven phase I metabolites and twenty-six phase II metabolites were detected. In most cases, their chemical structures were proposed based on recorded HR-MS/MS spectra. The exact masses of most abundant fragment ions are listed in Table S1 in Supplementary data. Following the classification used by Caspar et al. [31], for compound identification, the accurate precursor ion has to be detectable and the underlying HR-MS/MS spectrum has to fit with the reference spectrum. For detection, only the accurate precursor ion has to be detectable. In a case, when a reference MS/MS spectrum was not available, at least the main peaks in obtained HR-MS/MS spectrum were assigned to particular fragment ions. Herein, only the typical fragment ions used for the identification of the metabolites will be discussed in detail to illustrate the main fragmentation patterns.

The MS^2^ spectrum of 25CN-NBOMe (M1; PM at *m/z* 327.1709, M + H) showed similar fragmentation as previously described in metabolic studies of halogenated NBOMes, 25I-NBOMe [29,31,33] and 25B- and 25C-NBOMes [32]. The major FIs correspond to the cleavage of the methoxybenzyl moiety (at *m/z* 121.0653) and tropylium ion (at *m/z* 91.0548) formed after the loss of the methoxy group (-30.0105). The FI representing the 2C part bearing the amino group (*m/z* 207.1134) was not detected under the experimental conditions.

The most abundant metabolites were formed as a result of *O*-demethylation of either of the methoxy groups, hydroxylation at the aromatic ring, or even at the alkyl chain and a combination thereof. *N*-Demethoxybenzylation represented relatively little deployed metabolic pathway. Hereby, we also report the conversion of the cyano group to the corresponding amide or carboxylic acid. In all matrices, the most prevalent phase I metabolites were the same: mono- and *bis*-*O*-demethylated products, hydroxylated products, and 2C-CN together with its deamino-hydroxylated counterpart. To identify the exact positions of present functional groups and to confirm the proposed metabolic pathways, reference standards of the metabolites were synthesized.

### 2.2. Synthesis of Reference Standards

**Figure 1:**
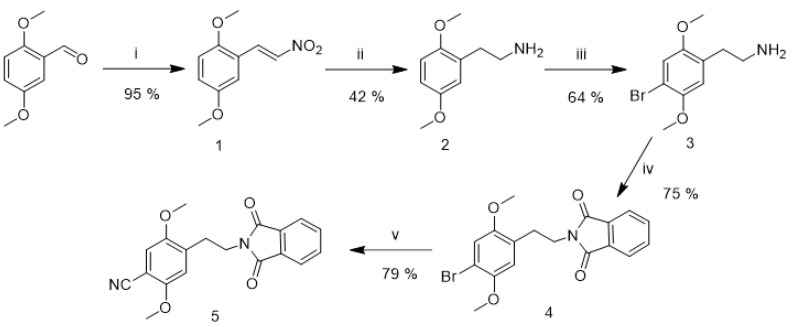
Synthetic route to the cyanide precursor. **i.** CH_3_NO_2_, IPA, EDDA; **ii**. Red-Al^®^, THF; **iii.** Br_2_, AcOH; **iv.** Phthalic anhydride, DMF, reflux; **v.** CuCN, DMF.

**Figure 2:**
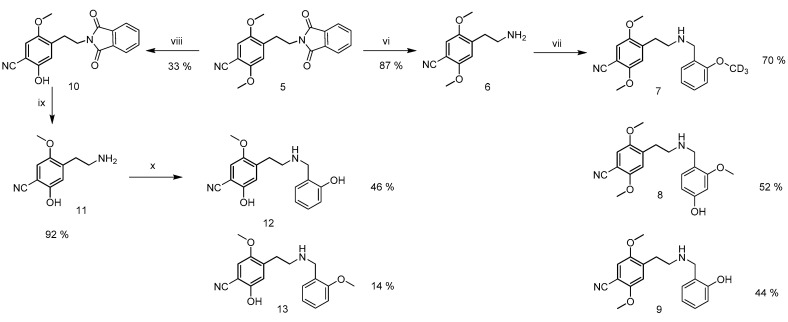
Synthesis of 25CN-NBOMe and its most abundant metabolites. **vi.** (a) NaBH_4_, IPA, H_2_O (b) AcOH, reflux; **vii**. (a) aldehyde (**7** 2-methoxy-d_3_-benzaldehyde; **8** 4-hydroxy-2-methoxybenzaldehyde; **9** salicylaldehyde), EtOH, (b) NaBH_4_; **viii.** BBr_3_ 1.1 eq., DCM, -79 °C to 20 °C; **ix.** (a) NaBH_4_, IPA, H_2_O, (b) AcOH, reflux; **x.** (a) aldehyde (**12** salicylaldehyde; **13** 2-methoxy-benzaldehyde), EtOH, Et_3_N, (b) NaBH_4_.

Based on the untargeted analysis, the most abundant metabolites were chosen, and their structures were proposed in accordance with their fragmentation in HR-MS/MS. The selected compounds were synthesized as reference standard materials (Figure 1 and Figure 2).

All synthesized standards (Figure 2, compounds **6**, **8**, **9**, **11**, **12,** and **13**) of expected major metabolites of 25CN-NBOMe were fully characterized (^1^H and ^13^C NMR, HR-MS, melting point) and their HR-MS/MS spectral data were compared to those obtained in the untargeted analysis.

### 2.3. LC-MS Analysis of the Most Abundant Metabolites

All synthesized metabolites were confirmed in rat urine and human liver microsomes, in *Cunninghamella elegans* culture medium *O*-demethyl-2C-CN (**11**) was not detected. Measured HR-MS/MS and MS^3^ data not only allowed structure confirmation but also provided deeper insight into 25CN-NBOMe’s metabolic fate and fragmentation patterns of individual metabolites.

Besides 25CN-NBOMe itself, two mono-*O*-demethylated metabolites were prepared as reference standards. The metabolite demethylated on the 2C moiety, *O*-demethyl-25CN-NBOMe (M9; PM at *m/z* 313.1552, M + H), was assigned as 2-hydroxy-5-methoxy-4-{2-[(2-methoxybenzyl)amino]ethyl}benzonitrile (**13**). It showed fragmentation typical for the whole group of metabolites bearing unchanged *N*-methoxybenzyl (NBOMe) moiety, with major FIs corresponding to the cleavage of this moiety (at *m/z* 121.0653) and to subsequently formed tropylium ion (at *m/z* 91.0548). The latter FI was accompanied by its hydrogenated counterpart (at *m/z* 93.07043) formed under certain experimental conditions. For the other *O*-demethylated metabolite, 25CN-NBOH (**9**) (M10; PM at *m/z* 313.1552, M + H), the prominent FI corresponded to the cleavage of hydroxybenzyl moiety (at *m/z* 107.0497). However, FIs representing the 2C part of the molecule showed comparable abundances. FIs were detected at *m/z* 207.1134, 190.0868, and 175.0633 representing protonated 2C-CN, an ion formed by loss of ammonia and by the subsequent loss of a methyl radical, respectively.

*O*,*O*-*bis*-demethylated metabolite, *O*-demethyl-25CN-NBOH (**12**) (M12; PM at *m/z* 299.1396; M + H) showed a similar fragmentation pattern with most abundant FIs registered at *m/z* 193.0977 and 176.0712 corresponding to 2C primary amine yielded by hydroxybenzyl cleavage and subsequent loss of ammonia. Cleaved methoxybenzyl ion was also detected at *m/z* 107.0497.

In MS^2^ spectra of the *N*-demethoxybenzylated metabolites 2C-CN (**6**; PM at *m/z* 207.1134, M + H) and its *O*-demethylated derivative **11** (M19, PM at *m/z* 193.0977), the main FIs were formed by amine shift (FI at *m/z* 190.0868 for 2C-CN and at *m/z* 176.0712 for *O*-demethyl-2C-CN), followed by loss of a methyl radical (FI at *m/z* 175.0633 for 2C-CN and at *m/z* 161.0477 for *O*-demethyl-2C-CN).

One of three detected mono-hydroxy-25CN-NBOMe isomers was assigned as 4′-hydroxy-25CN-NBOMe (M2, PM at *m/z* 343.1658) by comparison with the synthesized reference standard (**8**). Its fragmentation was typical for the whole group of compounds hydroxylated at the aromatic ring of NBOMe moiety, with the most abundant FIs corresponding to the cleavage of the modified NBOMe moiety (at *m/z* 137.0603) and to subsequently formed tropylium ion bearing the hydroxy group (at *m/z* 107. 0497). FIs representing the 2C part of the molecule were also present in the MS/MS spectrum and comprised ions detected at *m/z* 207.1134, representing 2C primary amine yielded by cleavage of modified NBOMe moiety, and at *m/z* 190.0868, formed by the subsequent loss of ammonia.

**Figure 3:**
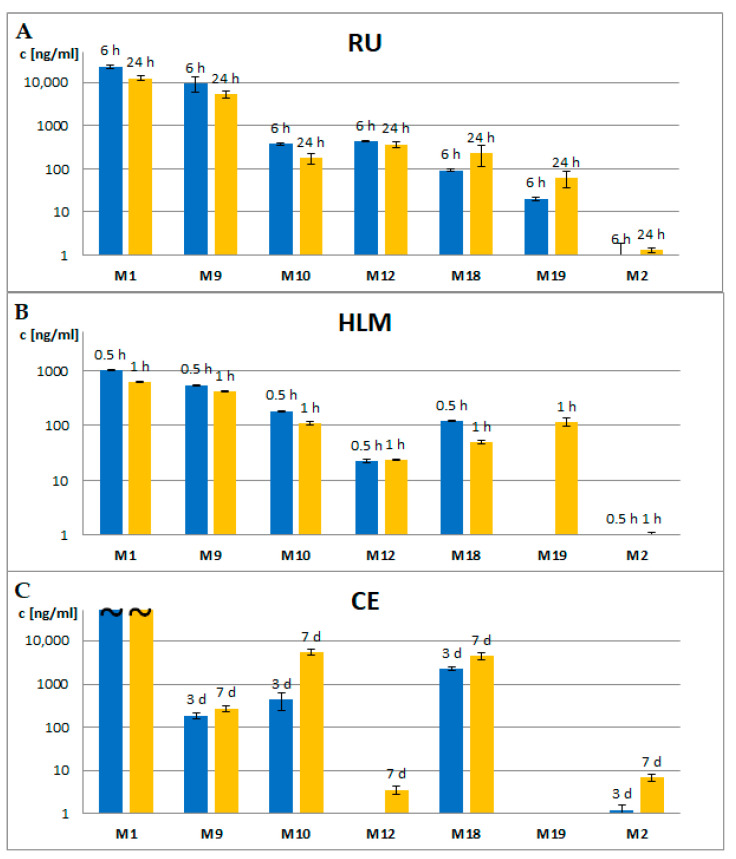
Comparison of concentration of individual 25CN-NBOMe metabolites in (**A**) rat urine (RU), (**B**) human liver microsome incubation (HLM) and (**C**) *C. elegans* culture media (CE) samples.

The most abundant first-order fragment ions of all synthesized metabolites were separated and further fragmented in a linear ion trap (LIT) of a triple quadrupole linear ion trap instrument. Both first and second-order fragments of individual 25CN-NBOMe metabolites are listed in Table 2. Based on identified transitions, a quantitative scan-free MS3 method [44] was developed to estimate the number of metabolites formed in vitro and in vivo experiments and to discuss the inter-species differences in 25CN-NBOMe metabolism. Results of quantification are graphically summarized in Figure 3.

### 2.4. Comparison of Metabolite Formation In Vitro and In Vivo and in Different Species

Varying results of metabolite formation were discovered in the three approaches used in this study. After subcutaneous injection of a relatively high dose of 25CN-NBOMe solution to rats, their urine was collected after 6 h and 24 h. The dose of 50 mg·kg^−1^ was selected according to the potency of related compounds, after it was proved to be safe for in vivo experiments using Modified Acute Toxicity assessment (OECD no. 423, 2001). Rat urine samples were generally rich in metabolites with twenty-four I phase and twenty-six II phase metabolites. The concentration levels of confirmed metabolites were also highest in this matrix. In vitro incubation of human liver microsomes produced similar phase I metabolites, just less in number and amount. In two cases, microsomes formed isomers hydroxylated in different positions compared to those found in rat urine (M4, M7). Even though monitoring of phase II metabolism in microsomes was not the original aim of this study, two glucuronidated metabolites (M9 G, M11 G) were found at low concentrations in microsomal samples. A uridine glucuronide transferase (UGT) is present in microsomes, as well as a limited amount of co-substrate (uridine 5′-diphospho-alpha-d-glucuronic acid) and co-factor (MgCl_2_) necessary for the optimal activity of the enzyme. Therefore, we presume the original supply of co-factors was consumed for the glucuronidation, and then the reaction catalyzed by UGT stopped, as the addition of critical co-factors was not included in our incubation protocol. *C. elegans* released only eight phase I metabolites formed by the most widespread metabolic transformations of 25X-NBOMe substances. From quantitative measurements, it was obvious that the extent of *O*-demethylation at the NBOMe part was reduced compared to other studied systems. On the other hand, the concentration of formed 2C-CN (M18) was relatively high and the amide derivative of parent drug M22, not found in any other matrix, was also detected in *C. elegans* culture medium.

### 2.5. Proposed Metabolic Pathways

Based on the metabolites identified in rat urine, human liver microsomes and fungus mycelium (summarized in Table 1), metabolic pathways of 25CN-NBOMe can be proposed.

Analogously to the previously published metabolic studies of halogenated NBOMes [29,32,33], 25CN-NBOMe undergoes the same major metabolic steps. The most abundant metabolites are the result of *O*-demethylation, *O-bis*-demethylation, and *O*-demethylation plus hydroxylation. *N*-Demethoxybenzylation represented relatively little deployed pathway. Hereby described is the newly found varying metabolism of cyano group to the corresponding amide or carboxylic acid, or its metabolic stability.

Phase I pathways were found to be mono-demethylation (M9, M10), bis-demethylation (M11, M12), and tris-demethylation (M17) of the methoxy groups, mono-hydroxylation (M2, M3, M4), *N*-demethoxybenzylation (M18), combinations of mono-demethylation with mono-hydroxylation (M8), bis-demethylation with mono-hydroxylation (M13, M14), or *N*-demethoxybenzylation with mono-demethylation (M19), followed by oxidative deamination (M20) and oxidation to a carboxylic acid (M21). Compared to the metabolism of analogous halogenated NBOMes [29,32,33], bis-hydroxylation, bis-hydroxylation with mono-demethylation, or the oxidation of the *N*-demethoxybenzylated 2C-CN to amide was not seen for 25CN-NBOMe.

Based on previously published discoveries in the metabolism of 25I-NBOMe, identifying hydroxylation in the benzylic para-position (4′-OH-25I-NBOMe) as the major metabolite [33], 4′-OH-25CN-NBOMe (**8**) was analogously synthesized as a reference standard. Although this metabolite was found (M2) and confirmed in our analysis, it is, however, not the most abundant isomer of benzylic mono-hydroxylation.

Several dehydrogenated-metabolites with the resulting double bond located at the 2C-moiety linker were found (M5, M6, M7, M15, M16). Dehydrogenation was previously detected in the metabolism of 25I-NBOMe and 25I-NBOH by Nielsen et al. [33] and of 25B- and 25C-NBOMe by Caspar et al. [32], while noting the dehydration could be possibly caused by artificial dehydroxylation under the analytical conditions of corresponding metabolites hydroxylated on ethyl-linker via unstable hemiaminals [32].

Two hydroxylated metabolites (M7 and M15) showed atypical chromatographic behavior, as they were eluted later than the parent compound and the appropriate *N*-oxide M6 on a reversed-phase column. In both cases, the metabolite’s polarity was reduced by the formation of an intramolecular hydrogen bond between the oxygens of the hydroxy group in the benzylic position and the methoxy group in NBOMe part of the molecule, causing the usual retention of the compounds.

Previously unreported is the metabolic fate of the cyano-functional group within the NBOMe scaffold. Based on the findings of in vitro metabolism of aromatic nitriles by rat liver subcellular fractions [45], two possible pathways were suggested. First is the direct conversion of the nitrile group to carboxylic acid and ammonia by nitrilase. The second pathway involves nitrile hydratase forming an amide followed by amidase forming carboxylic acid and ammonia. In our present study of the metabolism of 25CN-NBOMe, conversion of nitrile to amide was observed in rat urine and mycelium (metabolite M22 as a result of nitrile hydrolysis and M23 as a result of nitrile hydrolysis combined with *N*-demethoxybenzylation and *O*-demethylation), whereas conversion to carboxylic acid was detected only in the samples of rat urine (metabolites M24 combined with bis-demethylation and mono-hydroxylation and M25 in combined with *N*-demethoxybenzylation) (Table 1). The fungus species likely contain only nitrile hydratase, but not amidase, and metabolism is therefore finished at the amide stage. An important discovery is that the cyano group remained completely unmetabolized by human liver microsomes in our experiments, suggesting there are no enzymes to convert this functional group present.

Phase II metabolism was predominant in rat urine samples, where several metabolites were formed by glucuronidation (G), sulfation (S) and/or *N*-acetylation (Ac) of phase I metabolites. Two metabolites were detected only in their conjugated forms, namely oxo-2C-CN glucuronidate (M26 G) and sulfate (M26 G), and hydroxy-2C-CN *N*-acetyl (M27 Ac).

## 3. Materials and Methods

### 3.1. In Vitro Incubations with Human Liver Microsomes

Cryopreserved (−80 °C) primary human liver microsomes (kept in the dark prior to use), pooled from 50 donors equally representing a true population sample and fully characterized for major cytochrome P450 activities and select Phase II enzymes, were used in this study (purchased from Thermo Fisher Scientific, Waltham, MA, USA) and used according to the recommendation and protocol from the manufacturer [46]. The microsomes were slowly thawed on ice. Then, a reaction mixture was prepared from 163 µL of 100 mM phosphate-buffered saline (PBS; pH 7.4), 2 µL of 25CN-NBOMe to a final concentration of 10 µM, and 25 µL of 20 mg·mL^−1^ of liver microsomes. The mixture was preincubated for 10 min. at 37 °C in a water bath. After that, the reactions were initiated by the addition of 10 μL of freshly prepared 20mM nicotinamide adenine dinucleotide phosphate (NADPH) dissolved in 0.1M PBS. The samples were incubated for 0, 15, 30, 45, and 60 min at 37 °C with gentle agitation. At each time point, the reactions were terminated by the addition of 200 µL of ice-cold acetonitrile. Immediately after the quenching, the samples were frozen and kept at −80 °C till the MS analysis. Besides, four negative control samples were prepared: a no microsome sample, a no 25CN-NBOMe sample, a no NADPH sample, and a sample with heat-activated microsomes (pretreatment at 45 °C for 30 min). Besides, to verify unaffected microsome metabolism, a positive control, was prepared using 10 µM testosterone. The experiment was done in two independent replicates.

Commercially available human microsomes were used in this study. Human volunteers or donors, which requires the ethic committee or inform consent, were not involved.

### 3.2. In Vitro Metabolism by Cunninghamella Elegans

Modified protocol by Grafinger et al. [38] was used for the cultivation procedure. *C. elegans* (approx. 1 cm^2^) was inoculated on potato dextrose (PD) agar plates (12 g L^−1^ PDB, 5 g L^−1^ agar) (Formedium, Hunstanton, UK) in a 90-mm Petri dish and grown at 24 °C in the dark for 10 days. The mycelial mat from one plate (i.e., approx. 58 cm^2^ of mycelium) was harvested with forceps into 10 mL of PD medium (12 g·L^−1^ PDB) and vigorously shaken for 1 min. The homogenized mass was transferred into a 500 mL Erlenmeyer flask containing 35 mL of fresh PD medium. The mycelium was grown in a rotary shaker at 100 rpm at 28 °C in the dark for 3 days into a single large blob. Two controls—one containing PD medium and mycelium, the second containing only PD medium—were set up analogously. After three days, 1.5 mL of the medium from each of the three flasks was transferred to a plastic tube and stored at −20 °C until further analysis. 25CN-NBOMe (50 mg) was resuspended in 4 mL sterile water with the help of a vortex. The final concentration of 25CN-NBOMe solution was 270 µg·mL^−1^. In a sterile tube, 3 mL of PD medium was mixed with 2 mL of 25CN-NBOMe solution, or with 2 mL of sterile water as a control, and added to the respective mycelial culture in a flask, followed by 35 mL of fresh PD medium. The 3-day old culture of *C. elegans* (i.e., little less than 58 cm^2^ of mycelium) from PD agar plate was harvested with forceps, resuspended in 10 mL of fresh PD medium by vigorous shaking, and added to the mycelial culture. The cultures were mixed by swirling, and 1.5 mL of each was transferred to a plastic tube and stored at −20 °C until further analysis. Samples of the medium 24, 48, and 72 h after 25CN-NBOMe addition were drawn (1.5 mL of each culture transferred into a plastic tube) and stored at −20 °C until further analysis. Seven days after 25CN-NBOMe addition, all mycelia were harvested. Medium (approx. 45 mL) was poured into a sterile 50 mL tube, the mycelia were squished with sterilized forceps and transferred into a weighted 50 mL tube, and the rest of the medium poured into a new 50 mL tube. In the case of 25CN-NBOMe-PD control, approx. 50 mL of the medium was poured into the tube.

### 3.3. Animals

All animals were male outbred Wistar rats acquired from VELAZ (Prague, Czech Republic) weighing between 200–275 g at the beginning of testing. The rats were housed two *per* cage under a controlled temperature (22 ± 2 °C) and humidity (30–70%) with food pellets and water *ad libitum*. 25CN-NBOMe was dissolved in physiological solution with Tween 20, and administered in a dose of 50 mg·kg^−1^ subcutaneously (*s.c.*) in a volume of 2 mL·kg^−1^. After administration, the rats (*n* = 6) were placed in a metabolic chamber, and the urine was collected into a pre-cooled collector that maintained the temperature of −4 °C for the following 24 h. The collected urine was subsequently frozen and kept at −80 °C until the analyses.

All procedures were conducted following the principles of laboratory animal care of the National Committee for the Care and Use of Laboratory Animals (Prague, Czech Republic), and Guidelines of the European Union (86/609/EU). The protocol was approved by the National Committee for the Care and Use of Laboratory Animals (Prague, Czech Republic) under the number: 59449/2016-MZE-17214.

### 3.4. Synthesis and Characterization of the Most Abundant Metabolites

#### 3.4.1. General Information

Chemicals used for the synthesis were obtained from commercial sources (Sigma Aldrich, St. Louis, MO, USA and Acros Organics, Geel, Belgium). Hexane, DCM, and EtOAc were acquired from commercial sources and were used after distillation. Molecular sieves were used to dry solvents denoted as dry. Other commercial reagents and solvents were used without further purification. The reactions were monitored with the aid of thin-layer chromatography (TLC Silica gel 60 F254) and pre-coated reverse-phase gel plates (TLC RP-18 F254). All operations involving air-sensitive reagents were performed under an inert atmosphere of dry argon and dried solvents. Melting points were determined using a melting point apparatus (PGH Rundfunk-Fernsehen, Niederdorf, Germany), and they are uncorrected. NMR spectroscopy was performed in CD_3_OD or DMSO at room temperature using a Varian Gemini 300 MHz (^1^H NMR 300 MHz, ^13^C NMR 75 MHz) and an Agilent 400 MR DDR2 (^1^H NMR 400 MHz, ^13^C NMR 100 MHz). Preparative HPLC was performed using COMBIFLASH RF200 UV/VIS (Teledyne ISCO, Lincoln, NE, USA). An in-house made column was prepared by filling a cartridge with polar silica (Merck, Darmstadt, Germany) for normal phase HPLC. For reverse phase HPLC, the commercially available RediSep^®^ Gold C18 column (Teledyne ISCO) was used.

#### 3.4.2. Experimental Details

The synthetic route to the cyanide precursor is depicted in Figure 1. The preparation of amine **3** (2C-B) started with a Henry reaction between 2,5-dimethoxybenzaldehyde and nitromethane [47]. The nitrostyrene **1** was first reduced to amine **2** using Red-Al^®^ [48] and brominated with elemental bromine in AcOH [49], yielding the amine **3** as HBr salt. A phthalimide **4** was prepared by heating freebase **3** with phthalic anhydride in DMF [50]. Finally, a phthalimide **5** was prepared from **4** using CuCN [50]. Syntheses of the metabolites are shown in scheme Figure 2.

4-(2-Aminoethyl)-2,5-dimethoxybenzonitrile (**6**)

Phthalimide **5** (336 mg, 1 mmol) was suspended in 2-propanol (24 mL) and water (4 mL). The mixture was cooled to 0 °C and sodium borohydride (256 mg, 7 mmol) was added portion-wise for 5 min. The mixture was allowed to warm to room temperature and stirred overnight. The next day glacial acetic acid (4 mL) was added dropwise for 5 min and the solution was refluxed for 5 h. After cooling to room temperature, the solvent was evaporated in vacuo, and the residue was dissolved in 30 mL of water with NaOH (4 g) and extracted with DCM (3 × 10 mL). The DCM extracts were combined, acidified using HCl (2 mL, 1M), and extracted with water (3 × 10 mL). The aqueous phases were basified using NaOH (2 mL, 2M) and extracted with DCM (3 × 10 mL). The combined organic layers were dried over sodium sulfate, filtered, and evaporated *in vacuo* to provide 205 mg of crude phenethylamine **6** as a colorless oil which solidified upon standing in a fridge overnight. The hydrochloride salt was precipitated from dry Et_2_O and crystallized from 2-propanol and Et_2_O as a white powder. The final yield of hydrochloride salt was 211 mg (87%).

**m.p.** (HCl salt) 225–227 °C; **^1^H NMR** (400 MHz, CD_3_OD, HCl salt) δ 7.21 (s, 1H), 7.12 (s, 1H), 3.92 (s, 3H), 3.86 (s, 3H), 3.18 (t, J = 7.3 Hz, 2H), 3.04 (t, J = 7.4 Hz, 2H); **^13^C NMR** (101 MHz, CD_3_OD, HCl salt) δ 157.29 (s), 152.80 (s), 133.89 (s), 117.21 (s), 115.81 (s), 100.99 (s), 57.15 (s), 56.67 (s), 40.23 (s), 30.18 (s).

2,5-Dimethoxy-4-(2-{[2-(methoxy-*d*_3_)benzyl]amino}ethyl)benzonitrile (**7**)

A freebase phenethylamine **6** (103 mg, 0.5 mmol) was dissolved in EtOH (6 mL), 2-(methoxy-*d*_3_)benzaldehyde (77 mg, 0.55 mmol) was added, and the mixture was stirred for 3 h at room temperature. The mixture was cooled using an ice bath and sodium borohydride (76 mg, 2 mmol) was added. After stirring for 1 h, water (1 mL) was added to decompose the remaining sodium borohydride and the solvents were evaporated *in vacuo*. The residue was dissolved in 10 mL of water and extracted with DCM (5 × 5 mL). The organic phases were combined and the solvent was evaporated *in vacuo*. The remaining oil was dissolved in 10 mL of Et_2_O and HCl (1 mL, 1M) was added. The ethereal layer was extracted with water (3 × 10 mL). To the combined water layers NaOH (1 mL, 2M) was added and extracted using DCM (5 × 10 mL). The combined organic phases were dried over sodium sulfate, filtered, and evaporated *in vacuo* to provide 160 mg of a crude substance **7** as a colorless oil. The hydrochloride salt was precipitated from dry Et_2_O and crystallized twice from MeOH and Et_2_O as white needles. The final yield of hydrochloride salt was 128 mg (70%).

**m.p.** (HCl salt) 194–196 °C; **^1^H NMR** (400 MHz, CD_3_OD) δ 7.48–7.43 (m, 1H), 7.43–7.38 (m, 1H), 7.20 (s, 1H), 7.12 (s, 1H), 7.09 (d, *J* = 8.2 Hz, 1H), 7.02 (tt, *J* = 4.6, 2.3 Hz, 1H), 4.26 (s, 2H), 3.91 (s, 3H), 3.82 (s, 3H), 3.28–3.20 (m, 2H), 3.15–3.07 (m, *J* = 9.4, 6.1 Hz, 2H); **^13^C NMR** (101 MHz, CD_3_OD) δ 159.34 (s), 157.30 (s), 152.69 (s), 133.64 (s), 132.75 (d, *J* = 3.2 Hz), 122.01 (s), 120.24 (s), 117.13 (s), 115.87 (s), 115.74 (s), 112.08 (s), 101.09 (s), 57.16 (s), 56.71 (s), 55.87–54.93 (m), 47.95 (s), 47.48 (s), 28.77 (s).

4-{2-[(4-Hydroxy-2-methoxybenzyl)amino]ethyl}-2,5-dimethoxybenzonitrile (**8**)

A freebase phenethylamine **6** (102 mg, 0.5 mmol) was added and the mixture was stirred for 3 h at room temperature. The mixture was cooled using an ice bath and sodium borohydride (76 mg, 2 mmol) was added. After stirring for 1 h, water (1 mL) was added to decompose the remaining sodium borohydride and the solvents were evaporated *in vacuo*. The residue was dissolved in 10 mL of water and extracted with DCM (5 × 5 mL). The organic phases were combined, and the solvent was evaporated in vacuo. The remaining oil was dissolved in 10 mL of Et_2_O and HCl (1 mL, 1M) was added. The ethereal layer was extracted with water (3 × 10 mL). To the combined water layers, a concentrated solution of ammonia (1 mL, 25%) was added and the product was extracted using DCM (5 × 10 mL). The combined organic phases were dried over sodium sulfate, filtered, and evaporated *in vacuo* to provide 160 mg of a crude substance **8** as a colorless oil. The hydrochloride salt was precipitated from dry Et_2_O and crystallized twice from MeOH and Et_2_O as white fluffy needles. The final yield of hydrochloride salt was 99 mg (52%).

**m.p.** (HCl salt) 208–210 °C; **^1^H NMR** (400 MHz, CD_3_OD, HCl salt) δ 7.19 (s, 1H), 7.18 (d, *J* = 8.2 Hz, 1H), 7.09 (s, 1H), 6.50 (d, *J* = 2.2 Hz, 1H), 6.42 (dd, *J* = 8.2, 2.2 Hz, 1H), 4.14 (s, 2H), 3.90 (s, *J* = 9.2 Hz, 3H), 3.84 (s, 3H), 3.82 (s, 3H), 3.21 (dd, *J* = 9.6, 6.1 Hz, 2H), 3.07 (dd, *J* = 9.4, 6.0 Hz, 2H); **^13^C NMR** (101 MHz, CD_3_OD, HCl salt) δ 160.58 (s), 159.13 (s), 155.85 (s), 151.24 (s), 132.22 (s), 115.65 (s), 114.39 (s), 114.24 (s), 109.32 (s), 107.00 (s), 99.62 (s), 98.52 (s), 55.69 (s), 55.25 (s), 54.61 (s), 46.22 (s), 45.60 (s), 27.33 (s).

4-{2-[(2-Hydroxybenzyl)amino]ethyl}-2,5-dimethoxybenzonitrile (**9**)

Substance **9** was prepared from the phenethylamine **6** (103 mg, 0.5 mmol) and salicylaldehyde (67 mg, 0.55 mmol) in the same way as substance **8**. The crude substance **9** (105 mg) was isolated as a colorless oil. The hydrochloride salt was precipitated from dry Et_2_O and crystallized twice from MeOH and Et_2_O as white needles. The final yield of hydrochloride salt was 75 mg (44%).

**m.p.** (HCl salt) 198–201 °C; **^1^H NMR** (400 MHz, CD_3_OD, HCl salt) δ 7.35–7.26 (m, 2H), 7.20 (s, 1H), 7.11 (s, 1H), 6.94–6.87 (m, 2H), 4.24 (d, J = 6.5 Hz, 2H), 3.91 (s, 3H), 3.83 (s, 3H), 3.26 (dd, J = 8.9, 6.4 Hz, 2H), 3.11 (dd, J = 8.8, 6.5 Hz, 2H); **^13^C NMR** (101 MHz, CD_3_OD, HCl salt) δ 157.47 (s), 157.31 (s), 152.67 (s), 133.70 (s), 132.63 (s), 132.37 (s), 121.01 (s), 118.64 (s), 117.16 (s), 116.25 (s), 115.84 (s), 115.75 (s), 101.07 (s), 57.14 (s), 56.68 (s), 48.21 (s), 47.39 (s), 28.81 (s).

4-[2-(1,3-Dioxoisoindolin-2-yl)ethyl]-2-hydroxy-5-methoxybenzonitrile (**10**)

Phthalimide **5** (335 mg, 1 mmol) was dissolved in dry DCM (15 mL) under an inert atmosphere. The solution was cooled to −79 °C and BBr_3_ in DCM (1.3 mL, 1M) was added dropwise during 5 min. The mixture was allowed to gradually warm back to room temperature for 3 h and then stirred for 48 h. Then, the reaction was cooled using an ice bath, quenched with EtOH (2 mL), and diluted with DCM (30 mL). Saturated NaHCO_3_ solution (20 mL) was added to neutralize the HBr, layers were separated, and the water phase was extracted with DCM (2 × 20 mL). The combined organic phases were washed with NaHCO_3_ (20 mL), dried over sodium sulfate, filtered, and evaporated *in vacuo*. The residue was purified using flash chromatography on an in-house made column filled with polar silica (DCM–EtOAc, gradual elution). It was recovered 126 mg (37%) of starting substance **5.** Product **10** was further recrystallized from the 2-propanol-DCM mixture yielding 107 mg (33%).

**m.p.** 248–251 °C; **^1^H NMR** (600 MHz, DMSO) δ 10.39 (s, 1H), 7.87–7.80 (m, 4H), 7.05 (s, 1H), 6.75 (s, 1H), 3.79 (t, *J* = 6.6 Hz, 2H), 3.54 (s, 3H), 2.87 (t, *J* = 6.6 Hz, 2H); **^13^C NMR** (151 MHz, DMSO) δ 168.05 (s), 154.56 (s), 150.63 (s), 134.74 (s), 132.09 (s), 123.46 (s), 118.65 (s), 117.39 (s), 113.86 (s), 96.87 (s), 56.43 (s), 37.76 (s), 29.53 (s).

4-(2-Aminoethyl)-2-hydroxy-5-methoxybenzonitrile (**11**)

Phthalimide **10** (101 mg, 0.31 mmol) was suspended in 2-propanol (12 mL) and water (2 mL). The mixture was cooled to 0 °C and sodium borohydride (117 mg, 3.1 mmol) was added portion-wise for 5 min. The mixture was flushed with argon and stirred at room temperature overnight. The next day glacial acetic acid (2 mL) was added dropwise for 5 min and the solution was refluxed for 5 h. After cooling to room temperature, HCl (0.35 mL, 35%) was added and the solvent evaporated. Usually, an acid-base extraction would be used for workup. However, this method couldn’t be used due to the high solubility of **11** in water. Instead, the remaining boric acid was removed as trimethyl borate. MeOH (20 mL) was added and the solvent evaporated. This procedure was repeated two more times. The residue was dissolved in EtOH, and undissolved sodium chloride was filtered off. The EtOH solution was evaporated *in vacuo* and the residue was dissolved in 10 mL of distilled water. The mixture was washed with DCM (3 × 5 mL) and the water portion was evaporated, leaving behind 65 mg (92%) of a hydrochloride salt of phenethylamine **11** as a yellow powder.

**m.p.** (HCl salt) 215–219 °C; **^1^H NMR** (400 MHz, CD_3_OD, HCl salt) δ 7.08 (s, 1H), 6.88 (d, J = 3.6 Hz, 1H), 3.83 (d, J = 2.9 Hz, 3H), 3.21–3.08 (m, 2H), 3.01–2.90 (m, 2H); **^13^C NMR** (101 MHz, CD_3_OD, HCl salt) δ 155.94 (s), 152.00 (s), 134.03 (s), 119.61 (s), 117.72 (s), 114.78 (s), 99.17 (s), 56.55 (s), 40.26 (s), 30.04 (s).

2-Hydroxy-4-{2-[(2-hydroxybenzyl)amino]ethyl}-5-methoxybenzonitrile (**12**)

The hydrochloride salt of phenethylamine **11** (46 mg, 0.2 mmol), salicylaldehyde (37 mg, 0.3 mmol), and triethylamine (40 mg, 0.4 mmol) were dissolved in EtOH (2 mL) and the mixture was stirred under an inert atmosphere overnight. The mixture was cooled to 0 °C, sodium borohydride (38 mg, 1 mmol) was added and the stirring was continued for 1 h. The solvent was evaporated and MeOH (10 mL) and HCl (0.15 mL, 35%) were added. The boric acid was evaporated *in vacuo* as trimethyl borate. Further 10 mL of MeOH were added, and the process was repeated 2 more times to get rid of all the boric acid. The residue was dissolved in EtOH and undissolved sodium chloride was filtered off. The EtOH solution was evaporated *in vacuo* and the residue was dissolved in 10 mL of distilled water. The mixture was washed with DCM (3 × 5 mL) and the water portion was stirred with activated charcoal for 2 h. After filtering off the charcoal, the water was evaporated, leaving behind the hydrochloride salt of substance **12** as a light gray powder. The final yield of the hydrochloride salt was 31 mg (46%). The substance has darkened upon standing at room temperature for few days. Therefore, it is recommended to store it under an inert atmosphere in a fridge.

**m.p.** (HCl salt) 72–76 °C (decompose); **^1^H NMR** (300 MHz, CD_3_OD, HCl salt) δ 7.35–7.23 (m, *J* = 13.8, 7.3 Hz, 2H), 7.06 (s, 1H), 6.95–6.84 (m, 3H), 4.21 (s, 2H), 3.79 (s, 3H), 3.26–3.16 (m, 2H), 3.08–2.94 (m, 2H); **^13^C NMR** (101 MHz, CD_3_OD, HCl salt) δ 156.04 (s), 154.56 (s), 150.45 (s), 132.43 (s), 131.18 (s), 130.94 (s), 119.59 (s), 118.16 (s), 117.19 (s), 116.23 (s), 114.83 (s), 113.40 (s), 97.83 (s), 55.14 (s), 46.79 (s), 46.01 (s), 27.26 (s).

2-Hydroxy-5-methoxy-4-{2-[(2-methoxybenzyl)amino]ethyl}benzonitrile (**13**)

The substance **13** was prepared from the hydrochloride of phenethylamine **11** (38 mg, 0.17 mmol) and 2-methoxybenzaldehyde (23 mg, 0.25 mmol) in the same way as the substance **12**. The crude hydrochloride salt of substance **13** was further purified using chromatography on reverse phase silica pre-packed in RediSep^®^ Gold C18 column (MeOH—water, gradual elution). The final yield of the hydrochloride salt was 8 mg (14%).

**m.p.** (HCl salt) 193–196 °C; **^1^H NMR** (400 MHz, CD_3_OD, HCl salt) δ 7.49–7.41 (m, 1H), 7.38 (dd, *J* = 7.5, 1.5 Hz, 1H), 7.13–6.95 (m, 3H), 6.85 (s, 1H), 4.24 (s, 2H), 3.91 (s, 3H), 3.80 (s, 3H), 3.21 (dd, *J* = 9.2, 6.3 Hz, 2H), 3.01 (dd, *J* = 9.1, 6.4 Hz, 2H); **^13^C NMR** (101 MHz, CD_3_OD, HCl salt) δ 157.91 (s), 154.62 (s), 150.45 (s), 132.36 (s), 131.30 (s), 131.23 (s), 120.63 (s), 118.96 (s), 118.15 (s), 116.20 (s), 113.44 (s), 110.66 (s), 97.88 (s), 55.14 (s), 54.74 (s), 46.56 (s), 46.10 (s), 27.28 (s).

### 3.5. Sample Preparation

#### 3.5.1. Human Liver Microsomes

Internal standard (20 μL of 1 μg·mL^−1^ solution of 25CN-NBOMe-d_3_) was added to each liquid sample (200 μL). Samples were concentrated by evaporation of acetonitrile using speedvac (Hanil Modul 4080C) and ammonium bicarbonate solution was used to adjust their pH to 8.4 approximately. The resulting aqueous phase was extracted with 400 μL of ether twice, and combined organic extracts were evaporated to dryness. The dry residue was reconstituted in 200 μL of 5% (*v/v*) methanol in 0.1% formic acid and this solution was transferred to an HPLC vial.

#### 3.5.2. *C. elegans* Mycelium & Culture Medium

Internal standard (20 μL of 1 μg·mL^−1^ solution of 25CN-NBOMe-d_3_) was added to each media sample (200 μL) and ammonium bicarbonate solution was used to adjust the sample’s pH to 8.4 approximately. The resulting aqueous phase was extracted with 400 μL of ether twice, and combined organic extracts were evaporated to dryness. The dry residue was reconstituted in 200 μL of 5% (*v/v*) methanol in 0.1% formic acid and this solution was transferred into an HPLC vial.

*C. elegans* mycelium was thawed and cut into small pieces. Approximately 300 mg of wet material, 100 mg of bullets for homogenization (Next Advance ZrOB05—yttria-stabilized zirconium oxide beads, 0.5 mm dia.), and 1 mL of 0.1% formic acid were added to the weighted Eppendorf tube. Each sample was thoroughly vortexed and homogenized using the Bullet Blender Gold (Next Advance) at 4 °C (10 min, speed 8). Consequently, the samples were centrifuged for 10 min at 13,200 RPM at 4 °C (Eppendorf Centrifuge 5415 R), the resulting supernatants were separated and their pH was adjusted to 8.4 approximately by addition of ammonium bicarbonate solution. The aqueous phase was extracted with 400 μL of ether three times and combined organic extracts were evaporated to dryness. The dry residue was reconstituted in 300 μL of 5% (*v/v*) methanol in 0.1% formic acid and this solution was transferred to an HPLC vial.

#### 3.5.3. Rat Urine

A method for metabolomics studies of NPS in rat urine was adapted [51]. Collected urine samples (200 μL) were diluted with the same volume of 9% (*v/v*) methanol in 0.1% formic acid containing internal standard (200 ng·mL^−1^ of 25CN-NBOMe-d_3_) and thoroughly vortexed. Consequently, the samples were centrifuged for 10 min at 13,200 RPM at 4 °C (Eppendorf Centrifuge 5415 R). The resulting supernatants were transferred to HPLC vials.

### 3.6. LC-MS Analysis

#### 3.6.1. Untargeted Screening

An UltiMate 3000 LC system (Thermo, Waltham, MA, USA) coupled with a hybrid quadrupole time-of-flight TripleTOF 5600 mass spectrometer (AB Sciex, Framingham, MA, USA) equipped with an ESI ion source was used for information-dependent acquisition (IDA) analyses. Prepared samples were separated with a Kinetex C18 (3 × 50 mm, 2.6 µm) LC column (Phenomenex, Torrance, CA, USA). A gradient elution with 0.1% formic acid + 5 mM ammonium formate in water (mobile phase A) and 0.1% formic acid in methanol (mobile phase B) was applied with the following time profile: 0 min, 98:2 (A:B); 0.3 min, 98:2; 0.4 min, 96:4; 1 min, 90:10; 12 min, 0:100; 16 min, 0:100; 17.6 min, 90:10; 18 min, 98:2; 20 min, 98:2. The flow rate of the mobile phase was 200 μL·min^−1^ and column temperature was set at 30 °C.

The MS/MS apparatus was operating in high sensitivity positive mode. The applied parameters of electrospray ion source were: temperature 350 °C, curtain gas (CUR) 35 psi, nebulizer gas (GS1) 30 psi and heater gas (GS2) 40 psi; ion spray voltage floating (ISVF) was set to 4000 V. The data acquisition mode in the information-dependent acquisition (IDA) experiments was set to obtain a high-resolution TOF-MS and MS/MS scans, both over a mass range 50–800 *m/z*. Collision-induced dissociation was triggered by rolling collision energy. Raw data generated from IDA were evaluated using Sciex OS-Q version 1.5.0.23389 software.

#### 3.6.2. Human Liver Microsomes and *C. elegans* Samples

The analysis was performed using the UltiMate 3000 LC system (Thermo) coupled with the QTrap 6500 mass spectrometer (AB Sciex) equipped with a Turbo V ESI ion source. Prepared samples (injection volume 10 μL) were separated with a Luna Omega Polar C18 (2.1 × 100 mm, 3 µm) column (Phenomenex). A gradient elution with 0.1% formic acid + 5 mM ammonium formate in water (mobile phase A) and methanol (mobile phase B) was applied with the following time profile: 0 min, 98:2 (A:B); 2 min, 98:2; 12 min, 2:98; 14.5 min, 2:98; 15 min, 98:2; 20 min, 98:2. The flow rate of the mobile phase was 400 μL·min^−1^ and the column temperature was set at 30 °C.

The MS/MS apparatus was operating in positive mode. The applied parameters of electrospray ion source were: temperature 450 °C, curtain gas (CUR) 20 psi, nebulizer gas (GS1) 30 psi and heater gas (GS2) 30 psi; ion spray voltage was set to 5350 V. A scan-free MS^3^ (MS/MS/MS) method [44] was developed for screening of 25CN-NBOMe metabolites, the details are summarized in Table 3. For data acquisition and management, Analyst software version 1.63 and MultiQuant 3.0.3 were utilized (AB Sciex).

#### 3.6.3. Rat urine Samples

The analysis was performed using the MicroLC 200 Plus microflow LC system (Eksigent) coupled with the QTrap 6500+ mass spectrometer (AB Sciex) equipped with an Optiflow Micro ESI ion source. Prepared samples (injection volume 20 μL) were separated by a YMC-Triart C18 (0.3 × 150 mm, 3 µm) column (YMC) protected with a KrudKatcher UHPLC in-line filter. Gradient elution with 0.1% formic acid in water (mobile phase A) and methanol (mobile phase B) was applied with the following time profile: 0 min, 95:5 (A:B); 1 min, 95:5; 2.5 min, 5:95; 6 min, 5:95; 7 min, 95:5; 15 min, 95:5. The flow rate of the mobile phase was 7 μL·min^−1^ and column temperature was set at 22 °C.

The MS/MS apparatus was operating in positive mode. The applied parameters of electrospray ion source were: temperature 200 °C, curtain gas (CUR) 20 psi, nebulizer gas (GS1) 20 psi and heater gas (GS2) 20 psi; ion spray voltage was set to 5000 V. For screening of 25CN-NBOMe metabolites in urine samples, the previously described scan-free MS^3^ experiment was used, its experimental details are summarized in Table 1. For data acquisition and management, Analyst software version 1.7.1 and MultiQuant 3.0.3 were utilized (AB Sciex).

## 4. Conclusions

Our 25CN-NBOMe metabolism study in rat urine, human liver microsomes, and mycelium of *C. elegans* confirmed that it is metabolized through *O*-demethylation, hydroxylation, and *N*-demethoxybenzylation as the major pathways, analogously to published in vitro and in vivo studies on 25B-, 25C-, and 25I-NBOMe. However, a difference in the position of hydroxylation was detected. We also identified the fate of the nitrile group, which was hydrolyzed and/or oxidized to amid or carboxylic acid by mycelium and rats, respectively, but remained unchanged in the human liver microsomes. The most abundant metabolites may serve as biomarkers of the 25CN-NBOMe intoxication.

## Figures and Tables

**Table 1:** 25CN-NBOMe phase I and II metabolites, their protonated precursor ion exact masses and retention times (RT) detected in rat urine (RU), human liver microsomes (HLM), and *C.* *elegans* (CE). Data were measured with the UltiMate 3000 (Thermo) coupled to the TripleTOF 5600 (AB Sciex) LC-MS system.

No.	Metabolite	Precursor ion Exact Mass [*m/z*]	RT [min]	RU	HLM	CE
M1	25CN-NBOMe	327.1709	7.9	I	I	I
M2	hydroxy-25CN-NBOMe isomer 1	343.1658	7.4	I		
M3	hydroxy-25CN-NBOMe isomer 2	343.1658	7.3	I	I	I
M4	hydroxy-25CN-NBOMe isomer 3	343.1658	7.7		D	
M5	dehydro-25CN-NBOMe	325.1552	7.3	D	I	
M6	dehydro-25CN-NBOMe-*N*-oxide	341.1501	9.8	I		D
M7	dehydro-hydroxy-25CN-NBOMe	341.1501	10.1		D	I
M8	*O*-demethyl-hydroxy-25CN-NBOMe	329.1501	6.9	D	I	
M9	*O*-demethyl-25CN-NBOMe	313.1552	7.5	I	I	I
M10	25CN-NBOH	313.1552	7.6	I	I	I
M11	*O*,*O*-*bis*-demethyl-25CN-NBOMe	299.1396	6.8	I		
M12	*O*-demethyl-25CN-NBOH	299.1396	7.1	I	I	D
M13	*O,O*-*bis*-demethyl-hydroxy-25CN-NBOMe isomer 1	315.1345	5.8	D		
M14	*O,O*-*bis*-demethyl-hydroxy-25CN-NBOMe isomers 2, 3, 4	315.1345	6.1–6.8	D		
M15	*O*-demethyl-dehydro-hydroxy-25CN-NBOMe	327.1345	9.2	D		
M16	*O,O*-*bis*-demethyl-dehydro-25CN-NBOMe	297.1239	6.9	D		
M17	*O,O*-*bis*-demethyl-25CN-NBOH	285.1239	5.7	D		
M18	2C-CN	207.1134	5.7	I	I	I
M19	*O*-demethyl-2C-CN	193.0977	4.9	I	D	
M20	*O*-demethyl-deamino-hydroxy-2C-CN	194.0817	6.0	D		
M21	*O*-demethyl-deamino-carboxy-2C-CN	208.0610	5.7	I		
M22	2C-CONH_2_-NBOMe	345.1814	7.1			D
M23	*O*-demethyl-2C-CONH_2_	211.1078	4.0.	D		
M24	*O,O-bis*-demethyl-hydroxy-2C-COOH-NBOMe	334.1291	5.4	D		
M25	2C-COOH	226.1079	4.5	D		
M8I G	*O-*demethyl-hydroxy-25CN-NBOMe glucuronide isomer 1	505.1822	6.2	I		
M8 G	*O*-demethyl-hydroxy-25CN-NBOMe glucuronide isomer 2	505.1822	6.7	D		
M9 G	*O*-demethyl-25CN-NBOMe glucuronide	489.1873	6.9	I	D	
M11 G	*O,O*-bis-demethyl-25CN-NBOMe glucuronide	475.1717	6.1	I	D	
M13 G	*O,O*-bis-demethyl-hydroxy-25CN-NBOMe glucuronide isomer 1	491.1666	5.4	D		
M14 G	*O,O*-bis-demethyl-hydroxy-25CN-NBOMe glucuronide isomer 2	491.1666	6.2	D		
M16 G	*O,O*-bis-demethyl-dehydro-25CN-NBOMe glucuronide	473.1560	5.6	D		
M17 G	*O,O*-bis-demethyl-25CN-NBOH glucuronide	461.1560	5.4	I		
M19 G	*O*-demethyl-2C-CN glucuronide	369.1298	4.0	D		
M26 G	oxo-2C-CN glucuronide	397.1247	4.5	D		
M3 S	hydroxy-25CN-NBOMe sulfate	423.1226	7.4	D		
M8 S	*O*-demethyl-hydroxy-25CN-NBOMe sulfate	409.1069	6.9	I		
M9 S	*O*-demethyl-25CN-NBOMe sulfate	393.112	7.7	D		
M11 S	*O*,*O*-*bis*-demethyl-25CN-NBOMe sulfate	379.0964	6.9	I		
M12 S	*O*-demethyl-25CN-NBOH sulfate	379.0964	7.1	D		
M13 S	*O,O*-*bis*-demethyl-hydroxy-25CN-NBOMe sulfate isomer 1	395.0913	5.8	D		
M14 S1	*O,O*-*bis*-demethyl-hydroxy-25CN-NBOMe sulfate isomer 2	395.0913	6.3	D		
M14 S2	*O,O*-*bis*-demethyl-hydroxy-25CN-NBOMe sulfate isomer 3	395.0913	6.8	D		
M17 S1	*O,O*-*bis*-demethyl-25CN-NBOH sulfate isomer 1	365.0807	5.7	D		
M17 S2	*O,O*-*bis*-demethyl-25CN-NBOH sulfate isomer 2	365.0807	6.4	D		
M26 S	oxo-2C-CN sulfate	301.0494	5.0	I		
M19 Ac	*O*-demethyl-2C-CN *N*-acetyl isomer 1	235.1083	7.1	I		
M19 Ac	*O*-demethyl-2C-CN *N*-acetyl isomer 2	235.1083	7.3	I		
M19 Ac G	*O*-demethyl-2C-CN *N*-acetyl glucuronide	411.1404	5.9	I		
M19 Ac S	*O*-demethyl-2C-CN *N*-acetyl sulfate	315.0651	6.2	D		
M27 Ac	hydroxy-2C-CN *N*-acetyl	265.1188	5.6	D		

I = identified by matching precursor ion mass and recorded HR-MS/MS spectrum; D = detected based on the precursor ion exact mass only.

**Table 2:** 25CN-NBOMe and its most abundant metabolites, protonated precursor ions, characteristic MS*^2,^* and MS*^3^* fragment ions, retention times (RT), and detectability in rat urine (RU), human liver microsomes (HLM), and *C. elegans* culture media (CE). Data were measured with the UltiMate 3000 (Thermo) coupled to the QTrap 6500 (AB Sciex) LC-MS system.

No.	Target Metabolite	Precursor Ion [*m/z*]	MS^2^ Fragment Ions [*m/z*] ^1^	MS^3^ Fragment Ions [*m/z*] ^1^	RT [min]	Detected in Sample
**M1**	25CN-NBOMe	327	77 (1), 91 (37), 93 (13), 121 (100), 205 (2)	**121**: 77 (8), 91 (100), 93 (83), 121 (60) **205**: 175 (31), 190 (79), 205 (100)	8.3	RU, HLM, CE
**M2**	hydroxy-25CN-NBOMe 1 (**8**)	343	107 (8), 137 (100), 175 (1), 190 (30), 207 (30)	**190**: 163 (1), 175 (21), 190 (100) **207**: 147 (1), 160 (3), 163 (1), 175 (100)	7.8	RU, HLM, CE
**M9**	*O*-demethyl-25CN-NBOMe (**13**)	313	91 (14), 93 (6), 121 (100), 150 (7), 312 (9)	**91**: 65 (100), 91 (65), 93 (97) **121**: 77 (8), 91 (88), 93 (100), 121 (34)	7.9	RU, HLM, CE
**M10**	25CN-NBOH (**9**)	313	77 (44), 79 (50), 106 (100), 160 (5), 175 (78), 190 (98), 207 (85), 313 (7)	**190**: 160 (5), 175 (100), 190 (3) **207**: 190 (100), 175 (10), 207 (2)	8.0	RU, HLM, CE
**M12**	*O*-demethyl-25CN-NBOH (**12**)	299	77 (4), 79 (5), 107 (42), 161 (2), 176 (67), 193 (100), 299 (22)	**176**: 121 (1), 148 (1), 149 (2), 161 (16), 176 (100) **193**: 149 (2), 161 (14), 176 (100), 193 (93)	7.5	RU, HLM, CE
**M18**	2C-CN (**6**)	207	105 (5), 133 (6), 147 (7), 160 (23), 175 (100), 190 (85)	**175**: 104 (1), 132 (1), 160 (100), 175 (16) **190**: 117 (1), 147 (1), 160 (5), 175 (100), 190 (2)	6.3	RU, HLM, CE
**M19**	*O*-demethyl-2C-CN (**11**)	193	91 (2), 118 (3), 121 (2), 133 (4), 148 (10), 161 (92), 176 (100)	**161**: 132 (2), 160 (14), 161 (100) **176**: 148 (2), 149 (2), 161 (15), 176 (100)	5.5	RU, HLM

^1^ Relative intensity of fragment ion is given in brackets [%].

**Table 3:** Parameters of the developed scan-free MS^3^ quantification method—observed transitions and appropriate ion source parameters for individual metabolites.

	1st Precursor [*m/z*]	2nd Precursor [*m/z*]	Quantifier/Qualifier Ion [*m/z*]	CE ^1^ [eV]	DP ^2^ [V]
25CN-NBOMe	327.2	121.1	93.0	19	9
	327.2	205.1	190.1	15	42
*O*-demethyl-25CN-NBOMe (13)	313.2	121.1	93.0	21	30
	313.2	91.0	65.0	64	21
25CN-NBOH (9)	313.2	207.2	190.1	17	90
	313.2	190.1	175.1	21	17
*O*-demethyl-25CN-NBOH (12)	299.2	176.1	161.1	22	65
	299.2	193.1	176.1	17	60
2C-CN (6)	207.2	175.1	160.1	26	50
	207.2	190.1	175.1	14	30
*O*-demethyl-2C-CN (11)	193.1	176.1	161.1	15	15
	193.1	161.1	132.1	28	39
hydroxy-25CN-NBOMe (8)	343.3	207.2	190.1	12	36
	343.3	190.1	175.1	17	21
25CN-NBOMe-d_3_ (7)	330.2	124.1	96.0	24	22
	330.2	205.1	190.1	15	42

^1^ CE = collision energy; ^2^ DP = declustering potential.

## Data Availability

The source data available upon a reasonable request.

## Supplementary Materials

The following are available online at https://www.mdpi.com/article/10.3390/metabo11040212/s1, Table S1. List of 25CN-NBOMe phase I and phase II metabolites with recorded precursor ion exact mass (TOF-MS), the corresponding characteristic fragment ions with the calculated exact masses (HR-MS/MS), elemental compositions, and deviations of the measured from the calculated masses.
